# Exploring current physicians’ failure to communicate clinical feedback back to transferring physicians after transitions of patient care responsibility: A mixed methods study

**DOI:** 10.1007/s40037-020-00585-1

**Published:** 2020-06-08

**Authors:** Judith L. Bowen, Joseph Chiovaro, Bridget C. O’Brien, Christy Kim Boscardin, David M. Irby, Olle ten Cate

**Affiliations:** 1 Department of Medical Education and Clinical Sciences, Elson S Floyd College of Medicine, Washington State University, Spokane, WA USA; 2 Department of Medicine, Division of Hospital Medicine, Oregon Health and Science University, and Portland Veterans Affairs Healthcare System, Portland, OR USA; 3 Department of Medicine and Center for Faculty Educators, University of California San Francisco School of Medicine, San Francisco, CA USA; 4 Center for Research and Development of Education, University Medical Center Utrecht, Utrecht University, Utrecht, The Netherlands; 5 Center for Faculty Educators, University of California San Francisco School of Medicine, San Francisco, CA USA

**Keywords:** Clinical feedback, Patient care transitions, Communication, Clinical reasoning processes

## Abstract

**Introduction:**

After patient care transitions occur, communication from the current physician back to the transferring physician may be an important source of clinical feedback for learning from outcomes of previous reasoning processes. Factors associated with this communication are not well understood. This study clarifies how often, and for what reasons, current physicians do or do not communicate back to transferring physicians about transitioned patients.

**Methods:**

In 2018, 38 physicians at two academic teaching hospitals were interviewed about communication decisions regarding 618 transitioned patients. Researchers recorded quantitative and qualitative data in field notes, then coded communication rationales using directed content analysis. Descriptive statistics and mixed effects logistic regression analyses identified communication patterns and examined associations with communication for three conditions: When current physicians 1) changed transferring physicians’ clinical decisions, 2) perceived transferring physicians’ clinical uncertainty, and 3) perceived transferring physicians’ request for communication.

**Results:**

Communication occurred regarding 17% of transitioned patients. Transferring physicians initiated communication in 55% of these cases. Communication did not occur when current physicians 1) changed transferring physicians’ clinical decisions (119 patients), 2) perceived transferring physicians’ uncertainty (97 patients), and 3) perceived transferring physicians’ request for communication (12 patients). Rationales for no communication included case contextual, structural, interpersonal, and cultural factors. Perceived uncertainty and request for communication were positively associated with communication (*p* < 0.001) while a changed clinical decision was not.

**Discussion:**

Current physicians communicate infrequently with transferring physicians after assuming patient care responsibilities. Structural and interpersonal barriers to communication may be amenable to change. Clarity about transferring physicians’ uncertainty and desire for communication back may improve clinical feedback communication.

**Electronic supplementary material:**

The online version of this article (10.1007/s40037-020-00585-1) contains supplementary material, which is available to authorized users.

## Introduction

Transitions of responsibility for patient care are common in modern healthcare settings. As a consequence of several forces, including efficiency demands, patient safety, and specialization, physicians often work as site-based specialists in designated blocks of time [1–3]. These structures require *transferring* physicians, those initially responsible for clinical reasoning decisions, to transition patients to *current* physicians. Unlike temporary patient handovers where physicians subsequently return to care for the same patients, these transitions disrupt the transferring physician’s clinical reasoning processes. Outcomes of decisions they made before the transition may not become easily known unless follow-up occurs. These outcomes of prior decisions are a form of clinical feedback—information embedded in the clinical reasoning process as the patient’s condition evolves—that help physicians calibrate their future clinical decisions and learn [4–7]. While efforts to close this clinical feedback loop have been described, physicians may not seek outcome information about all their patients [6, 8, 9]. After-transition communication from current physicians back to transferring physicians could also close this clinical feedback loop, but the frequency of and factors associated with this communication are not well understood. If communication does not occur, opportunities to learn and improve future clinical reasoning processes may be lost [5].

Researchers have documented verbal[10] and non-verbal[11] communication challenges *during* patient care transitions and made recommendations for studying[12] and improving the quality of these transitions to address patient safety concerns [11, 13–17]. While communication about clinical outcomes may take place post-hoc for a small number of patients with unfortunate outcomes in the form of morbidity and mortality conferences[18], no studies address communication from current physicians back to transferring physicians *after* care transitions have occurred and when patients’ clinical trajectories have evolved. Factors associated with communicating clinical feedback about transitioned patients as part of physicians’ daily work have not been identified.

When viewed through the lens of situated cognition theory, interactions between individuals and their work environments are integral to learning from work performance such as caring for patients [19, 20]. Situated cognition posits that knowing and doing are inseparable because knowledge is embedded in the social, cultural, and physical contexts in which it occurs. From this perspective, learning occurs during activities in which individuals and context interact in ways that produce new knowledge, meaning, or insight. Thus, the activity of caring for patients with particular problems in clinical contexts with particular cultures, relationship expectations, and resources will shape learning [19]. In contexts where transitions of patient care responsibility occur, discontinuity is an important contextual dimension. Three conditions specific to the dimension of discontinuity may influence transferring physicians’ learning via a current physician’s decision to communicate back about a patient’s outcome. First, when the care transition occurs, current physicians may perceive transferring physicians to express uncertainty about diagnoses or management decisions for complex clinical cases and wish to assist with reducing that uncertainty via clinical feedback [21, 22]. Second, if transferring physicians express a desire to know what happened and request communication back, the current physician may then be more likely to share clinical feedback. Third, when current physicians change transferring physicians’ clinical decisions, anticipation that transferring physicians will be receptive to clinical feedback in these situations may be important [21, 23–25]. Thus, the very structures that require transitions of responsibility may also influence whether and how communication occurs, and what transferring physicians’ learn when transitions disrupt their clinical reasoning processes.

In order to better understand how to support transferring physicians’ learning in the context of discontinuity, we must first understand why current physicians do or do not communicate back to transferring physicians. Therefore, the purpose of this study is to clarify[26], in the context of actual practice, in what situations and for what reasons current physicians do or do not communicate with transferring physicians about transitioned patients for whom transferring physicians are no longer responsible.

## Methods

### Study design

We employed a convergent, mixed methods design, collecting both quantitative and qualitative data at the same time to develop a more comprehensive understanding of *current* physicians’ communication patterns and their rationales in the context of patient care transitions [27]. We used the qualitative data to amplify the quantitative data. We defined *patient care transition* as a formal transition of responsibility for patients that occurs when the transferring physician will no longer have any ongoing responsibility. We distinguish these formal transitions from handovers that occur at the end of shifts when covering physicians accept temporary responsibility and the primary physician returns the next day to resume responsibility for the same patients.

Several research questions guided our inquiry. For the quantitative strand of this study, we asked: 1) How often do current physicians communicate with transferring physicians about patients that transitioned between them? And, 2) What are the associations between communication and the conditions we hypothesized might influence whether or not current physicians communicate back to transferring physicians? We selected communication occurrence about a patient as the primary outcome variable. To explore associations with a communication occurrence, we selected three situational *conditions of interest* amenable to quantitative responses as shown in Tab. 1: a) when current physicians perceived transferring physicians’ uncertainty, b) when current physicians perceived transferring physicians’ request for communication, and c) when current physicians changed transferring physicians’ clinical decisions. For the qualitative strand, we asked: how do current physicians describe their rationales for communicating or not communicating back to transferring physicians (Tab. 1)? The institutional review boards at Oregon Health & Science University and the Portland Veterans Affairs Health Care System approved the study (IRB ID: STUDY00015374, May 16, 2018). The principal investigator (JB) obtained informed consent from all study participants. This work was carried out in accordance with the Declaration of Helsinki, including but not limited to, there being no potential harm to participants, that the anonymity of participants was guaranteed, and that informed consent was obtained from each participant.

**Table 1:** Conditions of interest (predictor variables) and the outcome variable, and associated structured interview questions for quantitative and qualitative strands

		Quantitative data	Qualitative data
	Definition	Structured interview questions	
*Condition of interest (Predictor variable)*			
Perceived transferring physicians’ uncertainty	The current physician perceived the transferring physician to express uncertainty about clinical decisions at the time of the care transition	Did the transferring hospitalist express uncertainty about the diagnosis or management for this patient at the time of transition? (yes/no)	
Perceived transferring physicians’ request for communication	The current physician perceived the transferring physician to request communication back about the case at the time of the care transition	Did the transferring hospitalist request communication back about this patient? (yes/no)	
Changed clinical decision	The current physician changed the transferring physicians’ diagnosis or management plan after the care transition	Did you change the patient’s diagnosis or management plan after the transition? (yes/no)	
*Outcome variable*			
Communication occurred	The current physician reported the status of communication with the transferring physician	Did you communicate back about this patient? (yes/no)	If yes, what was the primary reason for the communication? Who initiated the communication? What was the method of communication? If no, what was the primary reason communication did not occur?

### Setting

This study took place at two academic teaching hospitals. Participants were members of academic hospitalist group practices at one or the other hospital. Physicians in these work groups are variably assigned to the traditional teaching service or the clinical hospitalist service. The teaching service physician supervises patient care provided by teams of learners, including resident physicians, medical students, and physician assistant students. The clinical hospitalist service physician provides direct care for patients with only rare involvement with trainees. Work assignments for physicians are 1–2 weeks in duration. The transitions of interest occurred when the transferring physician transitioned patients to the current physician at the end of these 7‑ or 14-day work assignments.

### Participants

We recruited internal medicine physicians with established working relationships with members of their practice group who routinely transition patient care responsibility between each other. We invited physicians to participate via weekly email messages until no further responses were received for three consecutive weeks. Following consent, work assignments were used to contact current physicians to schedule interviews approximately 4 days after the participant (current physician) had received patient care responsibility from the transferring physician. We chose to collect data 4 days after the patient care transition based on the average length-of-stay in both hospitals, working under the assumption that most of the diagnostic and dispositional decisions would have occurred by then and could serve as potential sources of clinical feedback communication.

### Data collection

We collected data during brief structured interviews (on average, one minute per transitioned patient); closed-ended questions yielded categorical data for the quantitative analysis, open-ended questions yielded descriptive data for the qualitative analysis (Tab. 1). The principal investigator, JB, conducted all of the interviews while participants were still on service and responsible for patient care. Because work assignments varied, JB conducted one to six interviews per participant. Using their structured sign-out sheet to refer anonymously to patients received in transition, JB asked participants questions about each transitioned patient and recorded quantitative responses and field notes using data collection forms to assure consistent data collection (Tab. 1). When possible, field notes captured verbatim phrases and were recorded as such; these are reported with quotations marks. JB transcribed field notes after each interview and entered anonymized categorical and descriptive data about each transitioned patient into a spreadsheet.

### Analysis

#### Quantitative analysis

To assess the study’s effect on communication for participants interviewed more than once, we first calculated the communication frequency by interview number to monitor for changes in communication behavior over time. We found no increase in feedback communication during the study period[28]; therefore, we included all of the data in the analyses. We used two approaches to explore associations between communication occurrence and the three conditions of interest: 1) perceived uncertainty, 2) perceived communication request, and 3) changed clinical decisions. First, we used descriptive statistics to determine the overall communication rate and communication frequencies for each condition. Next, we examined the effect of each condition on the binary outcome, communication versus no communication. To account for the nested structure of the data, we conducted bi-variable mixed effects logistic regression analyses using each condition as a predictor variable (Stata SE 13.1 for Windows, Stata Corp, College Station, Texas). Then, all three predictors were included in the final multivariable mixed-effects logistic regression model to determine the relative contributions of each as predictors on communication outcome.

#### Qualitative analysis

Two researchers (JB and JC) independently analyzed all participants’ anonymized rationales for communication decisions from the interviews. JB is a physician and education researcher with previous experience as a physician on a traditional teaching service at one of the study sites but has no current work relationship with participants at either site. Her experience allowed for shared understanding of the colloquial terms participants used during the interviews. JC is an academic physician with current experience on both teaching and clinical hospitalist services at the other study site. This researcher’s insights about workflow facilitated timing and location of interviews. To address his potentially compromising role, he did not serve as a study subject nor conduct any interviews. Hospital identity was removed prior to JC’s involvement in the analysis. JB and JC developed an initial coding structure based on factors previously found to influence communication between current and transferring physicians [21]. Following directed qualitative content analytic procedures [29], we applied the following codes for the rationales provided: (1) case contextual (e.g. specific case details), (2) structural (e.g. work proximity), and (3) interpersonal (hierarchy, familiarity, relationship). Rationales not fitting these definitions were coded as (4) other. After independently coding a subset of rationales, we discussed findings, revised the codebook, and both researchers independently applied the codes to the entire data set derived from interview field notes. We negotiated differences through discussion. A third researcher, BO, an experienced medical education qualitative researcher, provided oversight to check interpretations throughout the analytic process. The final codebook is available as Supplemental Digital Appendix 1. We calculated occurrence rates for coded rationales to display frequencies [30]. In 2% of cases, we identified more than one rationale but included only the first for frequency calculations.

## Results

The principal investigator (JB) interviewed 21 Site 1 physicians (78% of eligible hospitalists) and 17 Site 2 physicians (71% of eligible hospitalists) over 3 months (May through July) in 2018. Twenty (53%) self-identified as male and 18 (47%) as female. JB conducted 94 interviews, 41 at Site 1 and 53 at Site 2. Each participant contributed an average of 2.5 interviews and discussed an average of 6.6 patients per interview. Participants transitioned 618 patient cases. As shown in Tab. 2, participants reported similar experiences by site, although current physicians changed transferring physicians’ clinical decisions more often at Site 1.

**Table 2:** Study site comparisons for numbers and frequency of communication occurrences and conditions of interest for all 618 patients transitioned during the study period in two academic hospitalist practices in 2018

	Site 1 *N*, %	Site 2 *N*, %	Total *N*, %
Patients transitioned during study period	260, 42%	358, 58%	618, 100%
Communication occurrences	39, 15%	68, 19%	107, 17%
*Frequency of condition of interest*			
Current physician perceived transferring physician’s uncertainty	69, 26%	84, 23%	153, 25%
Current physician perceived transferring physician’s communication request	23, 9%	25, 7%	48, 8%
Current physician changed transferring physician’s clinical decision(s)	95, 36%	74, 21%	169, 27%

Next, we report results for our primary outcome, when communication occurred, and associations of communication with the conditions of interest: a) perceived uncertainty, b) perceived communication request, and c) changed clinical decisions (Tab. 1.) We use qualitative results to elaborate why communication occurred. Then we report results when communication did not occur under the same conditions and use qualitative results to elaborate why communication did not occur.

### When communication occurred

Communication between current and transferring physicians occurred in 107 of 618 (17%) cases. Of these, the transferring physician initiated communication 55% of the time, and these communications occurred face-to-face 58% of the time. As shown in Tab. 3, communication was positively associated with all three conditions in the mixed effects bi-variable logistic regression analysis. However, in the final mixed effects multivariable logistic regression model, current physicians’ perceptions of transferring physicians’ uncertainty and request for communication were positively and significantly associated with communication occurrence but changing transferring physicians’ clinical decisions was not significantly associated with communication.

**Table 3:** Mixed effects logistic regression analysis examining the conditions associated with communication occurrence

	Mixed effects bi-variable logistic regression			Mixed effects multivariable logistic regression		
	OR	95% CI	*P*-value	OR	95% CI	*P*-value
Perceived uncertainty	6.1	3.7, 10.4	<0.001	3.0	1.6, 5.8	<0.001
Perceived communication request	41.4	15.8, 108.3	<0.001	22.2	8.3, 59.0	<0.001
Changed clinical decision	3.7	2.3, 6.3	<0.001	1.4	0.7, 2.7	0.290

Participants’ (current physicians’) rationales for communicating back under the three conditions of interest appear in Tab. 4. Rationales for most communication occurrences across all conditions involved case contextual factors. For example, current physicians provided clinical updates intended to “clarify,” “inform,” and “confirm.” Some rationales included elaborate descriptions, such as sharing reasons for why the patient was transferred to the intensive care unit for more aggressive treatment of refractory heart failure. In a few examples, interpersonal factors facilitated communication, such as knowing the transferring physician was “clinically curious”. Occasionally, participants used the word “collaborated” to describe working together on subsequent clinical decisions.

**Table 4:** Current physicians’ communication frequency and rationales for *communicating back* to transferring physicians for 107 out of 618 cases where communication occurred in two academic hospitalist practices in 2018

		Condition of interest					
		Perceived transferring physicians’ uncertainty		Perceived transferring physicians’ communication request		Changed transferring physicians’ clinical decisions	
		**Yes**	No	**Yes**	No	**Yes**	No
Communication occurred	**Yes**	*59, 9%*	48, 8%	*38, 6%*	69, 11%	* 50, 8%*	57, 9%
	No	97, 16%	414, 67%	12, 2%	499, 81%	119, 19%	392, 64%
Frequency of rationales by category for *communication*		*For all 59 communication occurrences when transferring physician expressed uncertainty (n, %)*		*For all 38 communication occurrences when transferring physician requested communication (n, %)*		*For all 50 communication occurrences when clinical decisions changed (n, %)*	
Case contextual factors		46, 78%		33, 87%		38, 76%	
Structural factors		2, 3%		0, 0%		3, 6%	
Interpersonal factors		8, 14%		4, 10%		7, 14%	
Other		3, 5%		1, 3%		2, 4%	
*Category*		*Examples of rationales for communication across 3 conditions*					
Case contextual factors^a^		– Provided update on clinical course – Clarified how current physician figured out complex medication refill history – Discussed challenging management situation – Updated and educated on rare diagnosis – Obtained more information about big picture to execute discharge – Explained reason for stopping antibiotics					
Structural factors^b^		– Ran into each other – Secondary to discussion of other patients					
Interpersonal factors^c^		– Discussed and co-managed, allowed nuanced tailoring of treatment – Provided reassurance that nothing was missed – Based on prior working relationship, knew she would want to know – Transferring physician wondered about current physician’s perception of his decision-making; conversation supportive, debriefed patient’s death					
Other^d^		– Kept everyone in the loop – Provided clinical update in front of physician assistants; pointed out work up recommended but not carried out – Communicated “thanks” back to transferring physician at family’s request					

^a^Rationales pertaining to the clinical case, including judgments about the learning value for the transferring physician

^b^Rationales pertaining to structural barriers including clinical information not yet available, no opportunity to communicate (i.e. on vacation), time constraints, or communication is scheduled in the future because the current physician will be transitioning patients back to the same physician

^c^Rationales pertaining to interpersonal factors including barriers related to hierarchy, familiarity with how the transferring physician will react, or no established relationship

^d^Rationales that did not fit into the above categories including references to the culture of communication or hedging efforts to communicate.

### When communication did not occur

Of cases where communication did not occur, the most common situation was that in which the transferring physician communicated confidence in the clinical assessment and the current physician subsequently agreed, made no changes to the transferring physician’s clinical decisions, and the clinical course turned out “as expected” (*n* = 362 out of 511, 71%). We explored further the 228 cases and their rationales where communication did not occur even though transferring physicians were perceived to have expressed uncertainty, and/or requested communication, and/or current physicians changed transferring physicians’ clinical decisions (Tab. 5).

**Table 5:** Current physicians’ frequencies and rationales for *no communication* back to transferring physicians for 228 out of 618 transitioned patients in two academic physician practices in 2018

		Condition of interest					
		Perceived transferring physicians’ uncertainty		Perceived transferring physicians’ communication request		Changed transferring physicians’ clinical decisions	
		**Yes**	No	**Yes**	No	**Yes**	No
Communication occurred	Yes	59, 9%	48, 8%	38, 6%	69, 11%	50, 8%	57, 9%
	**No**	*97, 16%*	414, 67%	*12, 2%*	499, 81%	*119, 19%*	392, 64%
Frequency of rationales by category for *no communication*		*For all 97 occurrences of no communication when transferring physician expressed uncertainty (n, %)*		*For all 12 occurrences of no communication when transferring physician requested communication (n, %)*		*For all 119 occurrences of no communication when clinical decisions changed (n, %)*	
Case contextual factors		38, 39%		1, 8%		38, 32%	
Structural factors		25, 26%		8, 67%		36, 30%	
Interpersonal factors		14, 14%		0, 0%		22, 19%	
Other		20, 21%		3, 25%		23, 19%	
CATEGORY		*Examples of rationales for* **no communication** *across 3 conditions*					
Case contextual factors^a^		– Mild shift in plan, course as expected – Expected clinical evolution, anticipatory guidance was clear – Nothing substantive, no major report to give back – Low yield learning opportunity – Clinically uninteresting – Final diagnosis was in the realm of expected					
Structural factors^b^		– Transferring physician on vacation, no opportunity to discuss – Too soon, just made the change today – Not enough information back to make the call – Waiting for the final path report – Too busy, diagnosis already on transferring physicians’ differential – Will sign patient back over to transferring physician					
Interpersonal factors^c^		– Expected transferring physician was chart stalking because he would be curious – It would be uncomfortable [for me] to tell my boss he was wrong – “Felt uneasy about our relationship” – Didn’t want transferring physician to feel judged about missing the issue – Because of defensive posture, I only communicate major issues – “She didn’t ask, I didn’t seek her out”					
Other^d^		– Planned communication not yet occurred – New work flow will [indirectly] come back to the group – Not a priority to communicate – Would potentially chat with transferring physician about this one – Didn’t think of it [communicating] – It’s how we operate [no communication culture]					

^a^Rationales pertaining to the clinical case, including judgments about the learning value for the transferring physician

^b^Rationales pertaining to structural barriers including clinical information not yet available, no opportunity to communicate (i.e. on vacation), time constraints, or communication is scheduled in the future because the current physician will be transitioning patients back to the same physician

^c^Rationales pertaining to interpersonal factors including barriers related to hierarchy, familiarity with how the transferring physician will react, or no established relationship

^d^Rationales that did not fit into the above categories including references to the culture of communication or hedging efforts to communicate.

As shown in Tab. 5, although we might have expected perceived uncertainty to serve as an invitation to communicate with transferring physicians, communication did not occur for most of these cases (97 of 156, Tab. 5, first condition.) When the transferring physician was perceived to invite communication, communication did not occur for some of these cases (12 of 50, Tab. 5, second condition). Current physicians changed the transferring physicians’ clinical decisions in 169 cases and communication did not occur in 119 of them (Tab. 5, third condition).

### Rationales for no communication across three conditions

Case contextual factors were frequent explanations in all cases of no communication. When providing these rationales, participants occasionally appeared to make judgments about the lack of learning value to the transferring physician (i.e. clinically uninteresting). We also noticed the rationale, “course as expected”, suggesting the current physician’s perception that the patient’s evolving clinical picture was consistent with the transferring physician’s anticipatory guidance, even though the current physician perceived uncertainty on the part of the transferring physician. We found rationales related to structural problems interfering with communication, including unavailable information at the time of the interview and lack of opportunity due to work schedules and vacations. Participants described barriers of hierarchy (“it would be uncomfortable [for me] to tell my boss he was wrong”) and challenging relationships with transferring physicians (“felt uneasy about our relationship”). Familiarity with transferring physicians’ reactions to clinical feedback, when negative (i.e. “defensive posture”), appeared to inhibit efforts to communicate. Knowing transferring physicians’ habits of “chart stalking” to find needed information led some to assume communication was not needed. Finally, we observed rationales we attributed to the workplace culture (coded as *other*)*.* For example, participants described communicating back as *not* “how we operate” in their group practice.

## Discussion

Researchers have studied communication challenges at the time of patient care transitions [11–15]. We explored transition communication from a different perspective—from current to transferring physicians to provide follow-up *after* transitions of responsibility had taken place. We observed a low communication frequency—only 17%—among physicians working in established hospitalist group practices. Conducting our study of discontinuity from the perspective of situated cognition illuminated several conditions that influence current physicians’ communication back to transferring physicians and may therefore impact learning and refinement of clinical reasoning. When communication did occur, it was associated with current physicians’ perceptions of transferring physicians’ clinical uncertainty and their requests for communication feedback. When communication did not occur, some conditions functioned as communication deterrents from the perspective of the current physician. Cases that turned out as expected were perceived to have low learning value. Structures such as opposite work schedules and competing patient care priorities deterred communication. Current physicians avoided communicating when relationship factors such as hierarchy and previous communication experiences were challenging. Lack of a communication culture also contributed to the low communication rate.

As we consider implications of communication back for transferring physicians’ learning, a lack of communication may not always be problematic. When the current physician’s clinical reasoning processes align with those of the transferring physician, uncertainty is not expressed, and nothing untoward happened to the patient, it may be that efforts to communicate back exceeded the perceived value of doing so. However, under conditions of transferring physicians’ uncertainty at the time of care transitions or current physicians’ changed clinical reasoning decisions after transitions, communicating back should be useful for improving care and learning [5].

Tracking patient outcomes is a learning habit of master clinicians [4, 31]. Experience of uncertainty from any source—evolution of the patient’s illness, clinical ambiguity, incomplete information, or anticipated but unknown changes to clinical decisions—leads some transferring physicians to seek clinical feedback [8]. We found that transferring physicians commonly initiated communication with current physicians. Although our study was not designed to explore transferring physicians’ reasons for initiating communication with current physicians, they may be motivated to reduce their uncertainty. Contrarily, it may be that physicians who transitioned off service simply engaged the current physician before the current physician had time to initiate the communication. Although not explicitly stated, a request for communication back could be interpreted as the transferring physicians’ desire for clinical feedback. In either case, transferring physicians may be signaling their receptivity to clinical feedback.

If transferring physicians seek follow-up on previous clinical decisions, either by chart review[9] or initiating communication, then what is the role of the current physician in providing clinical feedback? It would seem that the learning opportunities for transferring physicians most at risk of getting lost in discontinuous care settings are those where transferring physicians do not track patients’ outcomes, anticipating low learning value [8]. In these situations, current physicians may be the only source of clinical feedback that could improve care and learning. Our findings suggest two specific conditions where transferring physicians’ learning opportunities may have been lost because communication did not occur: when current physicians changed transferring physicians’ clinical reasoning decisions or transferring physicians expressed uncertainty at the transition. Only one of these, perceptions of expressed uncertainty, was significantly associated with communication. From a situated cognition perspective, transferring physicians’ expressions of uncertainty places responsibility on current physicians to provide clinical feedback to build shared knowledge and insights in ways that benefit patients.

What did we learn about conditions that influence communication and could be modified to improve learning in discontinuous care settings? Notably, case contextual factors were the driving force for most instances of both communication and non-communication. In some cases, current physicians made judgments about the informational value to transferring physicians. That clinical cases evolved and turned out “as expected” may suggest there is nothing left to learn. Physicians make such judgments based on their personal, situated interpretations of prior experiences, which likely differ between colleagues [7, 19]. Communication for the purpose of learning may correct erroneous or confirm effective reasoning processes. If left unaddressed, a lack of communication may unknowingly reinforce poor decisions and limit physicians’ abilities to more accurately predict future events [32]. Although more research is needed to understand these issues, our results suggest that clinical feedback communication might improve the quality of care patients receive in discontinuous care settings and should be a work expectation.

Structures posed common barriers to communication in the three conditions of interest in this study. Time constraints suggest physicians made trade-offs when communication did not occur. When communication cannot occur conveniently, it may require effort physicians feel they cannot afford to invest. The intent to communicate may simply get lost in the shuffle of competing demands. Work schedules facilitated and inhibited communication. Physicians transitioning patients between them work opposite schedules and are often unavailable during their ‘off’ weeks. Contrarily, physicians working opposite weeks are sometimes scheduled to transition back-to-back, creating a scheduled communication opportunity. Future investigations could explore ways to overcome these structural barriers to communication.

### Limitations

Our methodological choices place limits on how broadly we might speculate about our contribution. We asked participants to indicate if communication occurred with transferring physicians, suggesting our findings represented conscious communication decisions when perhaps they did not. We simultaneously collected data from current physicians about their communication decisions and their perceptions of the transferring physicians’ uncertainty and request for communication. It is possible their communication behavior influenced answers to these interview questions. We purposefully scheduled our interviews to include a time interval between transition and interview to allow the current physician’s clinical reasoning processes to take place. We relied on current physicians’ perceptions of transferring physicians’ uncertainty and request for communication. Future studies could explore both views of transition communication by 1) witnessing the actual communication at transitions, and 2) following up with both the transferring and current physicians.

## Conclusion

In clinical learning environments where frequent transitions of responsibility interrupt transferring physicians’ clinical reasoning processes, current physicians’ communication back to transferring physicians about evolving clinical outcomes may be an important source of clinical feedback. Knowing the outcomes of previous reasoning may facilitate learning and improve patient care. Yet, current physicians in this study communicated infrequently, raising questions about lost learning opportunities. Some of the structural and interpersonal barriers to communication we identified may be amenable to changes. We suggest physicians start with creating expectations for clinical feedback communication and refining their care transition communication processes to include clarifying transferring physicians’ uncertainty and desire for communication back, which may reduce some of the barriers to follow-up communication.

## Caption Electronic Supplementary Material

Supplemental Digital Appendix 1. Code book for directed content analysis of field notes
