# Supplementary material for: Exploring current physicians’ failure to communicate clinical feedback back to transferring physicians after transitions of patient care responsibility: A mixed methods study
Source: Perspect Med Educ. 2020 Jun 8;9(4):236–44. doi: 10.1007/s40037-020-00585-1 (PMC7459044; doi:10.1007/s40037-020-00585-1)
Supplement: Supplementary file 1 — Supplemental Digital Appendix 1. Code book for directed content analysis of field notes [file 40037_2020_585_MOESM1_ESM.docx]

Supplemental Digital Appendix 1. Code book for directed content analysis of field notes.

1. **Case Contextual factors** (defined as references to the clinical case, medical aspects of the case, including psychosocial aspects such as discharge planning)
   1. Clinical update (defined as any communication of clinical information)
   2. Course as expected (defined as the diagnosis and plan were carried forward and nothing unexpected happened; more a statement of fact about the case)
   3. Low learning utility (defined as clinically uninteresting, no substantive change, effort to communicate exceeds value of providing the information; infers a value judgment on the part of the current about the value of information to the transferring physician)
2. **Structural factors** (defined as any structural element in the learning / clinical care environment that facilitated or impeded communication, such as proximity or opportunity, time, scheduled communication because of the structure of work, or availability of clinical information that might serve as the basis for communication)
   1. Information not available (defined as the reason for communication is contingent upon clinical information that is not available or just became available without time for communicating in relationship to the timing of the interview)
   2. Communication scheduled (any reference to the current indicating that he/she would be transitioning patients back over to the same hospitalist as transferring physician, which creates a scheduled communication expectation between them and a convenient way to communicate; we do not know if the indicated patient was discussed nor the rationale at the time of the transition; this is distinguished from rationales of “planned communication”, which we coded as *hedging*, see below)
   3. No opportunity (defined as the inability to communicate because the transferring physician was on vacation, for example)
   4. Time constraints (any reference to time interfering with the decision to communicate)
3. **Interpersonal factors**
   1. Hierarchy (any reference to position power within the dyad, either direction)
   2. Familiarity (defined as knowledge of transferring physician, such as transferring hospitalist might be interested in clinical feedback or knowing how they might react to clinical feedback)
   3. Relationship (defined as having or not having a relationship that might influence communication)
4. **Other** (for any rationale that did not fit with the above definitions)
   1. For Communication events:
      1. Explicit educational opportunity (any rationale that explicitly stated for the purpose of education or learning)
      2. Idiosyncratic (just didn’t fit anything else and was case specific)
   2. For No communication events:
      1. Communication culture (defined as group norms or behaviors)
      2. Hedging (defined as rationale suggested communication would have value but was constrained, either still contemplating communicating or communicating indirectly)
